# PD-1、TIM-3、LAG-3、BTLA在结外NK/T细胞淋巴瘤中的表达及其预后价值

**DOI:** 10.3760/cma.j.issn.0253-2727.2021.07.012

**Published:** 2021-07

**Authors:** 璐 聂, 笑吟 刘, 荣军 马, 晓莉 袁, 丽 姜, 靖 杨, 爱侠 胡, 真 李, 尊民 朱

**Affiliations:** 1 河南省人民医院血液科，郑州 450003 Department of Hematology, Henan Provincial Hospital, Zhengzhou 450003, China; 2 河南省人民医院病理科，郑州 450003 Department of Pathology, Henan Provincial Hospital, Zhengzhou 450003, China; 3 河南省人民医院血液病研究所，郑州 450003 Institute of Hematology, Henan Provincial Hospital, Zhengzhou 450003, China

结外自然杀伤/T细胞淋巴瘤（ENKTL）是原发鼻腔最常见的一种侵袭性非霍奇金淋巴瘤（NHL），难治且易复发，患者生存差异大，也难以确定最佳预后因素，因此有必要探寻一些可靠标志物以尽早评估患者预后，并指导临床行个体化精准治疗。目前，针对免疫检查点（IC）的靶向治疗已使不少肿瘤患者获益，其中，表达于多种免疫细胞表面的程序性死亡因子1（PD-1）、T细胞免疫球蛋白黏蛋白分子3（TIM-3）、淋巴细胞活化基因3（LAG-3）及B和T淋巴细胞衰减因子（BTLA）常通过抑制免疫细胞效应，介导肿瘤细胞逃逸进而导致肿瘤的发生、发展[1]。但其在ENKTL中的表达及预后价值尚不明确。因此，本研究探索了PD-1、TIM-3、LAG-3及BTLA在ENKTL组织中的表达，并分析其对ENKTL患者预后的影响，旨在为ENKTL的免疫治疗探寻新靶点，进而改善患者预后。

## 病例与方法

1. 病例：以2013年11月至2018年12月于河南省人民医院病理科确诊、具有完整病例及随访资料的34例ENKTL蜡块标本作为观察组，随机收取9例鼻部炎症组织蜡块作为对照组。ENKTL的诊断符合文献[2]标准。入组标准：①组织标本均经手术切取且保存完好；②术前未经任何特殊药物治疗；③无其他严重基础性疾病；④未并发其他肿瘤；⑤确诊后至少行4个疗程规律治疗。本研究遵循《赫尔辛基宣言》，并通过了河南省人民医院伦理委员会的批准。

2. 临床资料及随访：收集ENKTL患者的临床资料，主要包括性别、年龄、确诊时间、B症状（发热、盗汗、体重减轻）、辅助检查结果、治疗方案及疾病进展时间等；综合评估患者病情，如ECOG评分、Ann Arbor分期、IPI评分、PINK评分等。

以电话随访为主。无进展生存（PFS）时间定义为患者确诊至发生疾病进展、死亡或失访的时间。总生存（OS）时间定义为患者确诊至任何原因所致死亡或者失访的时间。中位随访时间为30（3～71）个月。

3. 免疫组化实验：调取并修整上述ENKTL及鼻炎组织蜡块，用切片机将其切为4 µm的连续薄片并铺片，依次行脱蜡水化、抗原修复、内源性过氧化物酶阻断、滴加一抗二抗、显色、复染液复染、封片。一抗均购自英国Abcam公司，分别为Anti-PD-1（ab233117）、Anti-TIM-3（ab185703）、Anti-LAG-3（ab235907）、Anti-BTLA（ab181406）。阳性对照分别为肺癌组织、人扁桃体、人体小肠组织；阴性对照为ENKTL组织（一抗为磷酸盐缓冲液）。以上四种抗体主要定位于细胞膜上。最终结果由两名血液病理学专家综合高倍镜下染色强度和肿瘤阳性细胞占比行半定量判定，并据不同抗体做适当调整，再将数据二分类为阴性和阳性[3]。①染色强度评分：不着色0分，淡黄色1分，棕黄色2分，黄褐色3分；②阳性细胞占比评分：随机读取10个高倍镜视野（400×），每视野100个细胞，阳性细胞率（％）＝阳性细胞/观察细胞×100％；阳性细胞率<10％计0分，10％～<25％计1分，25％～<50％计2分，≥50％计3分。最后组织评分（H评分）为染色强度评分×阳性细胞占比评分，定义H评分≤3为阴性，H评分>3为阳性。

4. 统计学处理：应用SPSS 25.0软件行数据统计分析。分类资料用例数和百分数描述统计量；计数资料组间差异比较采用连续校正卡方检验或者Fisher精确检验；采用Spearman行相关性分析；生存描述采用Kalplan-Meier法，组间比较采用Log-rank检验。*P*<0.05为差异有统计学意义。

## 结果

1. 临床特征：在34例ENKTL患者中，男25例（73.5％），女9例（26.5％），中位年龄46（27～75）岁；据Ann Arbor分期，Ⅰ/Ⅱ期25例（73.5％），Ⅳ期9例（26.5％）；据PINK评分，低/中危27例（79.4％），高危7例（20.6％）；初诊伴B症状22例（64.7％），12例（35.3％）无B症状；原发于鼻腔外的有3例（8.8％），具体部位分别为喉部、扁桃体及舌部；所有患者EBV-EBER（+），1例（2.9％）患者乙肝病毒抗原（+）；接受GDP/GDP-L方案（吉西他滨+顺铂+地塞米松/GDP+左旋门冬酰胺酶或培门冬酶）联合放疗者18例（52.9％），接受CHOP/CHOP-L（环磷酰胺+吡柔比星+长春新碱+泼尼松/CHOP+左旋门冬酰胺酶或培门冬酶）联合放疗者16例（47.1％），比较两治疗组的一般临床特征，差异无统计学意义（*P*值均>0.05）。鼻炎对照组共9例，其中男7例，女2例，中位年龄51（27～68）岁。

2. 各蛋白在ENKTL组织中的表达：PD-1、TIM-3、LAG-3及BTLA均表达于细胞膜，部分抗体在胞质内可见弱染色。免疫组化结果示，四种蛋白均可不同程度地在ENKTL组织中表达（图1）。阳性率分别为50％（17/34）、64.7％（22/34）、44.1％（15/34）、91.2％（31/34）。

**图1:**
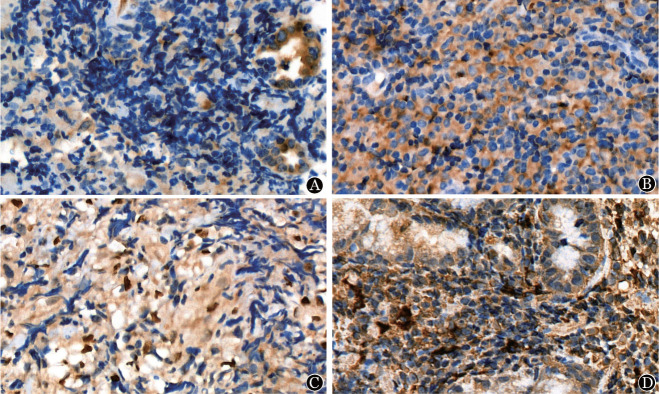
PD-1、TIM-3、LAG-3及BTLA蛋白在结外自然杀伤/T细胞淋巴瘤组织中的表达（400×） A：PD-1蛋白；B：TIM-3蛋白；C：LAG-3蛋白；D：BTLA蛋白

3. 各蛋白间表达相关性分析：PD-1、TIM-3、LAG-3和BTLA是免疫共抑制分子，故考虑其在ENKTL组织中的表达可能存在一定的关联。分析结果表明在34例ENKTL肿瘤组织中，PD-1和TIM-3的表达呈正相关（*r*＝0.492，*P*＝0.003）。

4. ENKTL与鼻部炎症组织中PD-1、TIM-3、LAG-3、BTLA表达水平的比较：分析结果示，TIM-3和LAG-3在两组间的表达差异有统计学意义（*χ*^2^＝6.203，*P*＝0.013；*χ*^2^＝4.310，*P*＝0.038）；而PD-1和BTLA表达在两组间差异无统计学意义（*P*>0.05）（表1）。

**表1:** 观察组和对照组PD-1、TIM-3、LAG-3、BTLA蛋白表达比较［例（％）］

组别	例数	PD-1阳性	TIM-3阳性	LAG-3阳性	BTLA阳性
观察组	34	17（50）	22（64.7）	15（44.1）	31（91.2）
对照组	9	1（11.1）	1（11.1）	0（0）	6（66.7）

*χ*^2^值		2.969	6.203	4.310	1.812
*P*值		0.085	0.013	0.038	0.178

5. 不同预后组蛋白表达水平比较：按患者完全缓解（CR）、复发（统计时剔除早期死亡病例）及死亡情况进行分组，进一步探讨ENKTL组织中四种蛋白的表达情况。结果显示：死亡组PD-1阳性率明显高于生存组（*P*＝0.038），而是否缓解、是否复发组间PD-1蛋白的表达差异无统计学意义（*P*>0.05）；TIM-3、LAG-3和BTLA三种蛋白按照不同结局分组后，组间蛋白表达差异均无统计学意义（*P*值均>0.05）（表2）。

**表2:** 不同预后组结外自然杀伤/T细胞淋巴瘤患者PD-1、TIM-3、LAG-3、BTLA蛋白表达阳性率比较［例（％）］

组别	例数	PD-1	TIM-3	LAG-3	BTLA
CR					
是	9	2（11.8）	6（27.3）	3（20.0）	8（25.8）
否	25	15（88.2）	16（72.7）	12（80.0）	23（74.2）
复发					
是	8	6（46.2）	6（35.3）	4（36.4）	8（33.3）
否	19	7（53.8）	11（64.7）	7（63.6）	16（66.7）
死亡					
是	17	12（76.6）^a^	9（40.9）	6（40.0）	14（45.2）
否	17	5（29.4）^a^	13（59.1）	9（60.0）	17（54.8）

注：CR：完全缓解。^a^两组比较，*P*<0.05

6. 与生存的相关性分析：据PD-1、TIM-3、LAG-3及BTLA表达情况，分为阴性组和阳性组。统计结果示PD-1阳性组的PFS率为26.7％，OS率为29.4％，阴性组的PFS和OS率分别为73.3％和70.6％，组间差异有统计学意义（*P*＝0.017，*P*＝0.027）（表3）。

**表3:** 不同PD-1、TIM-3、LAG-3、BTLA蛋白共表达情况下患者的估计生存时间（月）

组别	例数	无进展生存（95％*CI*）	*P*值	总生存（95％*CI*）	*P*值
PD-1			0.017		0.027
阴性	17	41.9（31.1～52.8）		53.8（40.4～66.6）	
阳性	17	22.8（13.7～32.0）		27.7（16.8～38.6）	
TIM-3			0.105		0.17
阴性	12	40.9（28.2～53.6）		51.7（36.0～67.3）	
阳性	22	26.6（17.9～35.3）		32.3（21.9～42.8）	
LAG-3			0.313		0.462
阴性	19	36.1（25.3～47.0）		45.8（32.4～59.3）	
阳性	15	27.6（17.0～38.1）		33.6（21.311～45.9）	
BTLA			0.193		0.128
阴性	3	56.0（56.0～56.0）		/	
阳性	31	29.3（21.2～37.3）		/	
PD-1+TIM-3			0.086		0.126
阴性	19	38.4（28.0～46.7）		49.1（36.1～62.2）	
阳性	15	24.6（14.7～34.5）		29.7（17.9～41.6）	
PD-1+LAG-3			0.061		0.117
阴性	25	37.3（28.0～46.7）		47.2（35.6～58.8）	
阳性	9	22.6（9.9～35.4）		27.2（12.9～41.6）	
PD-1+BTLA			0.017		0.027
阴性	17	42.0（31.1～52.8）		53.5（40.4～66.6）	
阳性	17	22.8（13.7～31.9）		27.7（16.8～38.6）	
PD1+TIM3+LAG3			0.161		0.282
阴性	26	36.0（26.7～45.3）		45.5（34.0～57.1）	
阳性	8	25.2（12.0～38.5）		30.1（15.2～45.1）	
PD1+LAG3+BTLA			0.061		0.117
阴性	25	37.3（28.0～46.7）		47.2（35.6～58.8）	
阳性	9	22.6（9.9～35.4）		27.2（12.9～41.6）	
TIM3+LAG3+BTLA			0.123		0.191
阴性	23	37.4（27.5～47.2）		47.3（35.1～59.5）	
阳性	11	25.1（13.5～36.7）		29.8（16.5～43.1）	
四蛋白共表达			0.161		0.282
阴性	26	36.0（26.5～45.3）		45.5（34.0～57.1）	
阳性	8	25.2（11.9～38.5）		30.1（15.2～45.1）	

注：/：无法预估

四种IC在多种疾病中存在共表达现象，因此同时分析在不同蛋白共表达情况下患者的预后情况，结果显示仅PD-1和BTLA共表达时，阳性组（PD-1和BTLA共表达）与阴性组（PD-1和BTLA均不表达，或者其一表达）PFS率、OS率差异具有统计学意义（*P*＝0.017，*P*＝0.027）。

## 讨论

肿瘤的发生、发展和机体的免疫状态密切相关，近年来，免疫治疗成为肿瘤治疗领域的研究热点。而ENKTL作为NHL中的一种少见亚型，具有高度侵袭性，患者起病常处于早期阶段、临床表现不典型，且预后较差。本研究探索PD-1、TIM-3、LAG-3和BTLA能否成为ENKTL潜在的干预靶点。

PD-1（CD279）是一种Ⅰ型跨膜球蛋白，是免疫球蛋白超家族（IgSF）中的重要成员，主要以与配体结合的方式对T细胞的免疫调节功能起抑制作用[4]。PD-1常表达于活化T、B、NK细胞及肿瘤浸润的淋巴细胞（TIL）表面[4]–[5]，而其主要配体程序性死亡因子-配体1（PD-L1）常表达于肿瘤细胞表面[6]。本研究表明PD-1在ENKTL组织中高表达，与其他研究结果一致[7]，但与在鼻部炎症组织中的表达相比，差异无统计学意义（*χ*^2^＝2.965，*P*＝0.085）。按不同结局分组，ENKTL患者死亡组PD-1阳性率显著高于存活组（*P*＝0.038）。生存分析结果也提示PD-1表达显著影响患者的PFS时间（*P*＝0.017）和OS时间（*P*＝0.027）。当前由美国食品和药品管理局批准的以PD-1为靶点的抑制剂主要有Pembrolizumab和Nivolumab，2017年Kwong等[8]应用Pembrolizumab治疗复发难治的NK/T细胞淋巴瘤（NKTL），CR率高达70％，2018第3版NCCN指南将Pembrolizumab纳入了NKTL的挽救治疗方案中。以上均表明PD-1可作为ENKTL免疫治疗的有效靶点。

本研究发现在ENKTL组织中，PD-1和TIM-3的表达呈正相关（*r*＝0.492，*P*＝0.003）。二者同为免疫共抑制分子，也可于卵巢肿瘤、肝炎相关性肝癌等疾病中共表达[9]–[10]。有研究发现，针对PD-1和TIM-3的单克隆抗体在人类乳腺癌移植物中上调其他IC基因的表达，表明阻断一个IC可以上调替代的IC，并可能导致肿瘤细胞逃避抗肿瘤免疫的补偿机制[11]。TIM-3也是一种Ⅰ型跨膜糖蛋白，属于TIM家族，通过与其天然配体半乳凝素-9（Gal-9）相结合发挥免疫调节作用[13]。其常表达于T细胞、树突细胞、NK细胞表面[12]–[13]，也有报道称TIM-3可表达于胃癌、黑色素瘤、小细胞肺癌、卵巢癌等的肿瘤细胞表面[10],[12]–[15]。近年来研究表明，TIM-3在急性髓系白血病、骨髓增生异常综合征、淋巴瘤等血液系统疾病中表达，且与不良预后相关[16]–[18]。我们发现TIM-3在ENKTL中的表达比例较鼻炎患者高（*χ*^2^＝6.203，*P*＝0.013），与其他研究结果一致[18]，但对ENKTL患者的生存影响不大（*P*>0.05），与Feng等[18]的研究结论不一致，可能与实验单染技术限制，阳性细胞掺杂正常免疫细胞相关。TIM-3的靶向抑制剂Ipilimumab已使不少黑色素瘤患者获益[19]，也有望成为ENKTL免疫治疗的靶点。

LAG-3（CD223）是IgSF中的一种跨膜蛋白，其染色体定位及结构均与CD4分子相似[20]。LAG-3主要表达于活化T和NK细胞的表面，常与主要组织相容性复合物-Ⅱ（MHC-Ⅱ）相结合而参与机体的负性免疫调节。近来有研究表明纤维蛋白原样蛋白1（FGL1）是其另一种重要的功能配体，FGL1-LAG-3通路是一种独立的肿瘤免疫逃避机制和免疫治疗的潜在靶点[21]。LAG-3和PD-1被证实在多种肿瘤中存在共表达，且对二者联合阻断的免疫疗法也有较好的前景[10],[21]–[22]，本研究表明二者共表达率为36％（9/25），但相关性并不显著（*P*＝0.315）。LAG-3在ENKTL中的表达与鼻炎患者相比，差异有统计学意义（*χ*^2^＝4.310，*P*＝0.038）。但其对患者的生存影响并不显著（*P*>0.05），与Feng等[18]的研究结果一致。

BTLA（CD272）也是IgSF的一种Ⅰ型跨膜糖蛋白，可不同程度地表达于T、B、NK、树突细胞等免疫细胞膜表面[23]，主要通过与配体疱疹病毒入侵介质（HVEM）相结合而减弱淋巴细胞的活化，进而下调免疫反应[24]–[25]。BTLA可在肺癌、黑色素瘤等多种恶性肿瘤的外周血免疫细胞、TIL及组织中表达[26]–[28]。在血液系统疾病中，BTLA常过表达于慢性淋巴细胞白血病及小淋巴细胞淋巴瘤[25]，Hobo等[29]发现阻断BTLA-HVEM通路，于行异基因造血干细胞移植术后的白血病患者，其次要组织相容性抗原（MiHA）特异性T细胞的增殖较阻断PD-1/PD-L1显著。本实验结果表明BTLA在ENKTL组织中的阳性率高达91.2％，与鼻炎患者相比差异无统计学意义（*P*>0.05），尚不能证实其对ENKTL的预后有影响。

四种IC均通过与其配体结合参与抑制T细胞活化、T细胞耗竭（常共表达PD-1、LAG3和TIM3）[30]，在多种疾病中起协同作用，但分子机制尚不明确，这也是今后我们要进一步研究的方向。靶向IC阻断去除T细胞活化的抑制信号，是针对可能导致细胞癌变的重要环节，其协同作用使免疫检查点抑制剂（ICI）在ENKTL中联合治疗成为可能。ICI抗肿瘤活性高、不良反应相对可控，我们期待更多的研究证实联合治疗的疗效，以期使更多的ENKTL患者获益。

总之，本研究证实ENKTL组织可不同程度地表达PD-1、TIM-3、LAG-3和BTLA，且蛋白间存在共表达，PD-1或PD-1/BTLA显著影响患者的生存时间。但本研究因样本量有限、免疫组化单染色技术不能很好地区分瘤细胞和免疫细胞，以及其他一些混杂因素可能致结果存在偏倚，未来尚需结合其他实验技术行多中心、大样本、前瞻性的研究以进一步探索IC在ENKTL中的预后价值。
